# The effects of YKL-40 on angiogenic potential of HUVECs are partly mediated by syndecan-4

**DOI:** 10.7150/ijms.55406

**Published:** 2021-10-15

**Authors:** WeiJun Sun, Qi Xue, Yan Zhao, Jianlei Zheng

**Affiliations:** 1 Department of Neurosurgery, Zhejiang Provincial People's Hospital, People's Hospital of Hangzhou Medical College, Hangzhou 310014, Zhejiang, China.; 2 Department of Cardiology, Zhejiang Provincial People's Hospital, People's Hospital of Hangzhou Medical College, Hangzhou 310014, Zhejiang, China.

**Keywords:** Migration, Signaling pathways, Syndecan-4, Tube formation, YKL-40

## Abstract

**Background:** YKL-40, a secreted glycoprotein, has a role in promoting tumor angiogenesis through syndecan-1 receptor. Syndecan-4 is a member of syndecan family. However, the effects of YKL-40 on migration and tube formation of human umbilical vein cells (HUVECs) mediated by syndecan-4 receptor are unknown.

**Materials and methods:** HUVECs were transfected with lentivirus encoding syndecan-4 short hairpin (sh) RNAs (lenti-synd4 shRNAs) and the efficiency of transfection was measured using qRT-PCR and western blotting. The effects of recombinant protein of YKL-40 on migration and angiogenesis of HUVECs adjusted by syndecan-4 were determined by wound healing and tube formation assay. The expressions of protein kinase Cα (PKCα) and extracellular signal regulated kinases (ERKs) 1 and 2 (ERK1/2) in HUVECs were measured using western blotting.

**Results:** The mRNA and protein expression of syndecan-4 were significantly decreased in HUVECs successfully transfected with lenti-synd4 shRNAs. Lenti-synd4 shRNAs remarkably inhibited the migration and tube formation of HUVECs stimulated by recombinant protein of YKL-40. The levels of PKCα and ratio of p-ERK1/2 to ERK1/2 in HUVECs were also decreased by down-regulating syndecan-4.

**Conclusion:** The effects of YKL-40 on migration and tube formation of HUVECs are partly inhibited by knock-downing syndecan-4 through suppressing PKCα and ERK1/2 signaling pathways.

## Introduction

YKL-40 (also known as human cartilage glycoprotein-39) is a 40 kDa heparin- and chitin-binding glycoprotein, which has been known to play an important role in cell migration, proliferation and angiogenesis 1,2. Recently, angiogenesis is regarded as a characteristic for vulnerable plaques 3. Immature neovessels in atherosclerotic plaque may contribute to intraplaque hemorrhage and inflammatory cells infiltration, so plaque neovessels were considered as a critical factor for progression and destabilization of atherosclerosis 4. In clinical practices, it has been reported that elevated serum YKL-40 was associated with acute coronary syndrome and cardiovascular morbidity and mortality 5-7. Hence, It is supposed that increased serum YKL-40 as a biomarker for adverse prognosis in cardiovascular diseases may be related to its role in atherosclerotic instability.

Syndecans (SDCs) are a family of heparan sulfate proteoglycans, including four mammalian syndecans, from syndecan-1 to syndecan-4, which play a critical role in cell adhesion, migration, proliferation and angiogenesis 8,9. The physiological and pathological function of SDCs are fulfilled by interacting with a great number of ligands including insoluble matrix collagens, associated glycoproteins and interleukins. Moreover, syndecans affect cell function via interacting with its adjacent membrane receptors such as integrin and fibroblast growth factor receptor (FGFR) 10,11. All syndecans are shown to carry similar molecular structure of heparan sulfate chains, and heparan sulfate is regarded as a physiological ligand for YKL-40 9,12. It was reported that YKL-40-induced tumor angiogenesis was dependent on the interaction via YKL-40 and membrane receptors syndecan-1 as well as integrin αvβ3 13. Different from the other three syndecans, syndecan-4 has a more widespread tissue distribution, and it was abundantly expressed in HUVECs 14. However, whether the angiogenic potential of YKL-40 in HUVECs is regulated by syndecan-4 is unknown, so the present study is to explore the effects of YKL-40 on migration and tube formation of HUVECs and potential signal pathways of YKL-40 mediated by syndecan-4.

## Materials and Methods

### Cell culture

HUVECs were purchased from Shanghai Institutes for Biological Sciences, Chinese Academy of Sciences (Shanghai, China). HUVECs were grown in DMEM (Gibco, USA) with 10% fetal bovine serum (FBS) (Thermo-Fisher Scientific, Waltham, USA) supplemented with 1% endothelial cell growth supplement (ECGs) (ScienCell Research Laboratories, Carlsbad, CA, USA) and 1% penicillin-streptomycin solution (Invitrogen, Carlsbad, CA, USA) and were incubated at 37 °C in a humidified atmosphere containing 5% CO2. HUVECs in the present study were divided into control (HUVECs without additional treatment), YKL-40 group (HUVECs stimulated with 100 ng/ml YKL-40, Cat No: 11227-H08H, Sino Biological Inc. China), YKL-40+lenti-synd4 group (HUVECs transfected with lentivirus encoding syndecan-4 shRNAs plus 100 ng/ml YKL-40) and YKL-40+lenti-null group (HUVECs transfected with lentivirus carrying scramble shRNAs plus 100 ng/ml YKL-40).

### The expression of syndecans in HUVECs and construction of lentiviral vector carrying syndecan-4 shRNAs

At first, we analyzed the gene expression of syndecan 1 to syndecan 4 in HUVECs. Gene expression level of syndecans was normalized to that of endogenous control, and the relative expression of target genes in HUVECs was calculated according to the 2^-ΔΔCt^ method. The primers for syndecans and glyceraldehyde-3-phosphate dehydrogenase (GAPDH) were described in Table 1. Then we designed three couples of short hairpin RNAs (shRNAs) and they were transfected into human embryonic kidney (HEK)293 cells. The shRNAs with most powerful inhibition to syndecan-4 expression were selected for subsequent experiments. A lentiviral vector silencing syndecan-4 expression was constructed as below. Briefly, The sequences used for shRNAs in this study are as follows: shRNAs (targeting syndecan-4): 5'-GCAGGAATCTGATGACTTTGATTCAAGAGATCAAAGTCATCAGATTCCTGCTTTTTT-3'; shRNAs (scramble): 5'-GCACCCAGTCCGCCCTGAGCAAATTCAAGAGATTTGC TCAGGGCGGACTGGGTGCTTTTT-3'. They were subsequently cloned into pLent-U6-GFP-Puro. Infectious viruses were produced by cotransfecting the lentiviral vector and packaging constructs (PMD2G and PSPAX2) into 293FT cells at passage 2-3 in medium with DMEM, 10% FBS, 2 mM L-glutamine and 1% penicillin-streptomycin solution. Infectious lentivirus particles were harvested at 72 hours after transfection. The final virus titres were approximately 5.0×10^8^ TU/mL, and the whole process of lentivirus construction was supplied by Shandong ViGene Biosciences (Jinan, Shandong, China). HUVECs at passage 2 were successfully infected with corresponding virus at a multiplicity of infection (MOI) of 30. At last, cells transfected with the lentiviruses were further selected for 48 hours in medium containing 2 ug/mL puromycin (Sigma-Aldrich, St. Louis, MO, USA) until the final stable cell clones were harvested and verified for following studies.

### The effect of lenti-synd4 shRNAs interference verified by qRT-PCR and western blotting

The total RNA was extracted from stable transfectants of HUVECs with TRIzol Reagent from Thermo Fisher Scientific (Waltham, MA, USA), and the RNA concentration was measured at 260 nm/280 nm absorbance ratio. The total RNA was reverse-transcribed to cDNA using a high capacity cDNA Reverse Transcription Kit from Genecopoeia (Maryland, USA). qRT-PCR was carried out to evaluate the effect of lenti-synd4 shRNAs on syndecan-4 expression with SYBP Premix Ex Taq TM from Takara (Shiga, Japan) following the manufacturer's instructions. In addition, the cells were harvested and the inhibition effect of lenti-synd4 shRNAs on membrane protein of syndecan-4 (Cat No: AF2918-SP, R&D Company, USA) was further measured with western blotting. Furthermore, the combination site of YKL-40 on the location of cell was evaluated with immunofluorescence (Cat No: 250114, anti his-tag mAb, Zen-bioscience, Chengdu, China).

### Wound healing assay

HUVECs at 95% confluence were plated at a density of 3×10^5^ cells/well in 6-well plates and cultivated in DMEM with ECGs medium and 1% FBS at 37 °C overnight. The monolayer of HUVECs was scratched with a sterile pipette tip to form wound gaps. Cells were then washed to remove debris by PBS and incubated with or without 100 ng/ml YKL-40 at 37 °C to induce cell migration for 24 hours and 32 hours. Images from two different scratch areas in each culture well were systematically obtained using a light microscope (magnification, ×40) equipped with Leica Application Suite program (Leica Microsystems, Switzerland). The rate of migration was measured as the ratio of closure area to initial wound: migration area (%) is equal to (A_0_-A_n_)/A_0_×100%. A_0_ means the area of initial wound area and A_n_ represents remaining area of wound at corresponding observation point. The difference of migration area at 24 hours and 32 hours relative to that at 0 hour was measured.

### Tube formation assay

Tube formation assay was performed to evaluate angiogenic ability of HUVECs. Briefly, the cells of control, YKL-40 group, YKL-40+lenti-synd4 group and YKL-40+lenti-null group were pretreated in medium containing 10% FBS and 1% EGCS for 24 hours or 48 hours, then were seeded into a 96-well plate at a density of 5×10^3^cells/well coated with Matrigel matrix (Cat No: 356230, BD Biosciences, CA, USA) for 6 hour incubation at 37 °C. Capillary tube structures were observed and representative images were captured using Leica Application Suite program (magnification, ×40). Cell were first stained with Calcein-AM (Cat No: C8950, Beijing Solarbio Science & Technology Co., Ltd, China), then the total tubule length, and the number of nodes, tubules and junctions representing the capacity of angiogenesis were determined using Image J software with automated Angiogenesis plug-in package 15.

### Western blotting analysis

The four groups of cells grown in 60 mm dishes were cultured according to corresponding medium for 24 hours or 48 hours. Then, they were lysed using Radio-Immunoprecipitation assay (RIPA) lysis buffer from Thermo Fisher Scientific (Waltham, MA, USA) at 4 °C for 30 minutes, and subjected to 12000 rpm centrifugation at 4 °C for 5 minutes. The protein concentration was measured by BCA protein assay Kit from Thermo Fisher Scientific (Waltham, MA, USA). The lysates were separated by 12% sodium dodecyl sulfate-polyacrylamide gel electrophoresis (SDS-PAGE), then transferred to polyvinylidene fluoride (PVDF) membranes and blocked with 5% skim milk. The blots were analyzed with antibodies according to the manufacturer's instructions and visualized by peroxidase and an enhanced chemiluminescence system from Pierce Biotechnology (Waltham, MA, USA). The corresponding antibodies were used in the present study including anti-p-ERK1/2 and anti-ERK1/2 (Cat No: YP1197, Immuno Way, USA; Cat No: ab65142, Abcam, Cambridge, UK, respectively); anti-PKCα and anti-β-actin (Cat No: ab32376 and ab8227, Abcam, Cambridge, UK, respectively). Goat anti-rabbit IgG secondary antibody (Cat No: ab205718, Abcam, Cambridge, UK).

### Statistical analysis

Data analysis was performed with SPSS13.0 (SPSS, Inc., Chicago, IL, USA) software. Data were expressed as mean ± standard deviation. Comparisons between two groups were evaluated by t-test. A p-value less than 0.05 was considered statistically significant.

## Results

### Syndecan-4 expression in HUVECs and interference effect of Lenti-synd4 shRNAs

**Figure 1:**
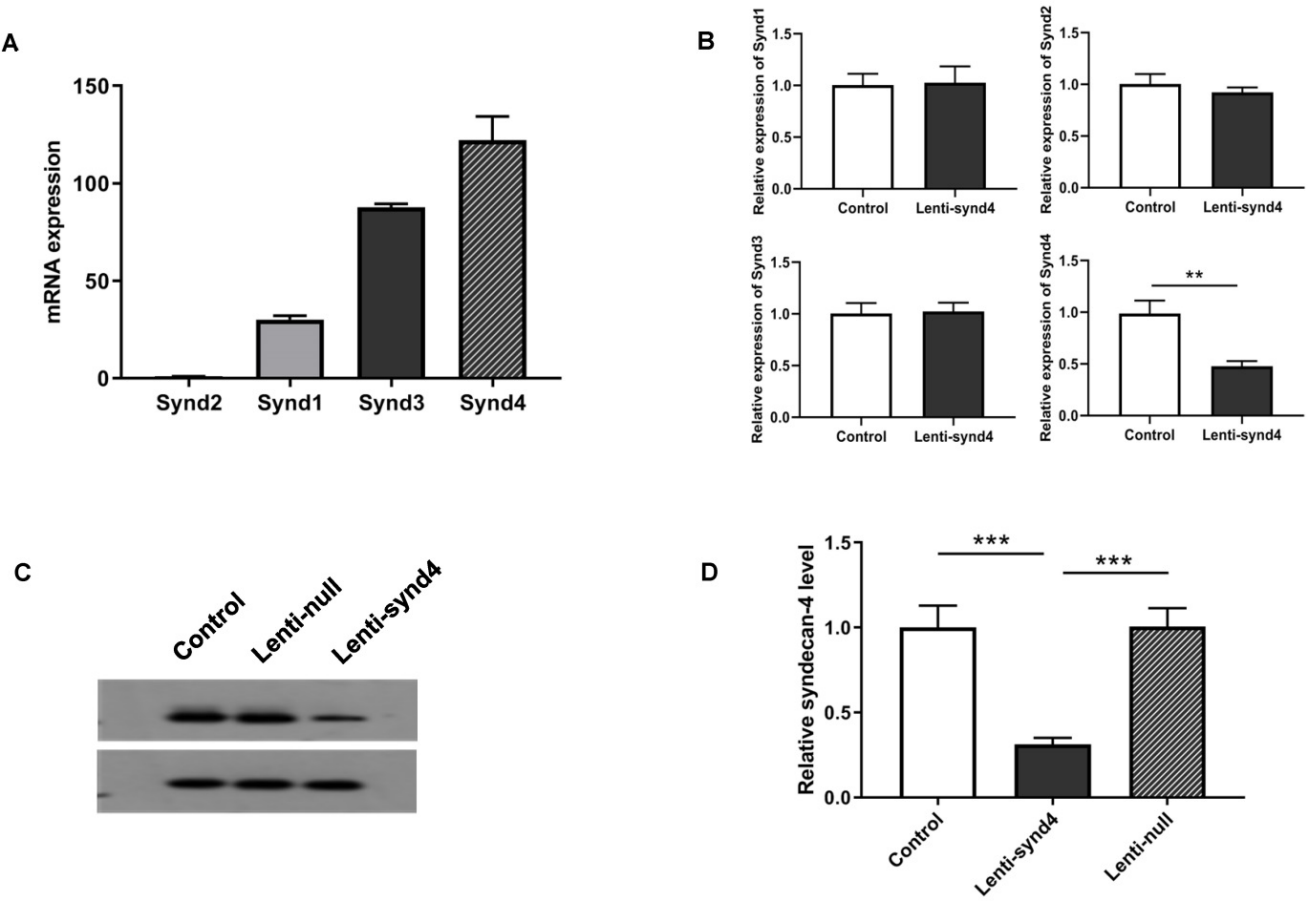
** The mRNA expression from syndecan-1 to syndecan-4 in HUVECs as well as Gene and protein expression of syndecan-4 from HUVECs after being transfected with lenti-synd4 shRNAs.** The mRNA expression of syndecan family in HUVECs (**A**). The interference effect of lenti-synd4 shRNAs on mRNA expression of syndecans in HUVECs (**B**). Membrane receptor of syndecan-4 on HUVECs was measured with western blotting among control, lenti-synd4 shRNAs group and lenti-null group (**C, D**). Data were presented as mean ± S.D. (n=3 per group). *** *p*<0.001 and **p<0.01.

**Figure 2:**
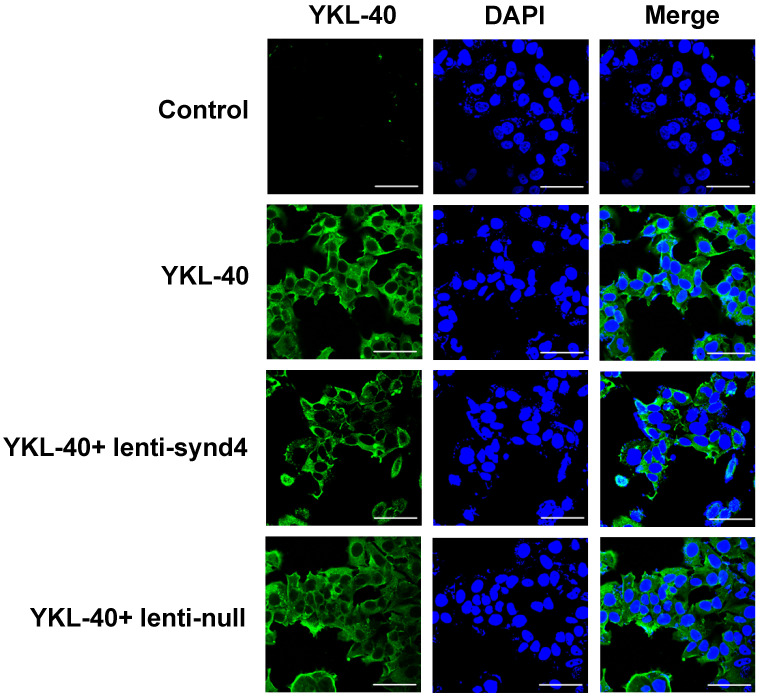
** The location site of YKL-40 in HUVECs.** With the aid of anti his-tag antibody, we found that YKL-40 was internalized into cytoplasm of HUVECs through immunofluorescence assay. Notably, lenti-synd4 shRNAs decreased the exogenous YKL-40 entering cytoplasm (the scale bar=50um, 400×magnification).

We demonstrated the syndecan-4 was the highest expression among the four syndecan family in HUVECs; however, the expression of syndecan-1 was relatively lower than the other three syndecans (Figure 1A). In fact, It has been shown that the expression of syndecan-4 was rather abundant in different endothelial cells 14,16. After HUVECs were successfully transfected with lentivirus, we found mRNA expression of syndecan-4 was remarkably decreased in HUVECs with lenti-synd4 shRNAs relative to control HUVECs (p<0.01). However, the mRNA levels of syndecan-1 to syndecan-3 had not been affected by lenti-synd4 shRNAs (Figure 1B). Moreover, the membrane receptor expression of syndecan-4 on HUVECs was further performed with western blotting. The data from the Figure 1C-D showed the syndecan-4 expression on the cell membrane of HUVECs was also remarkably decreased in lenti-synd4 shRNAs group, in comparison to the lenti-null group (p<0.001). On the other hand, with anti his-tag antibody, we demonstrated that the location of YKL-40 in HVUECs was cytoplasm (Figure 2). In addition, we found that lenti-synd4 shRNAs inhibited YKL-40 from entering cytoplasm, which may be associated with the reduction of syndecan-4 on cell membrane and syndecan-4 as a potential receptor for YKL-40.

### The effect of lenti-synd4 shRNAs on the migration of HUVECs treated with YKL-40

**Figure 3:**
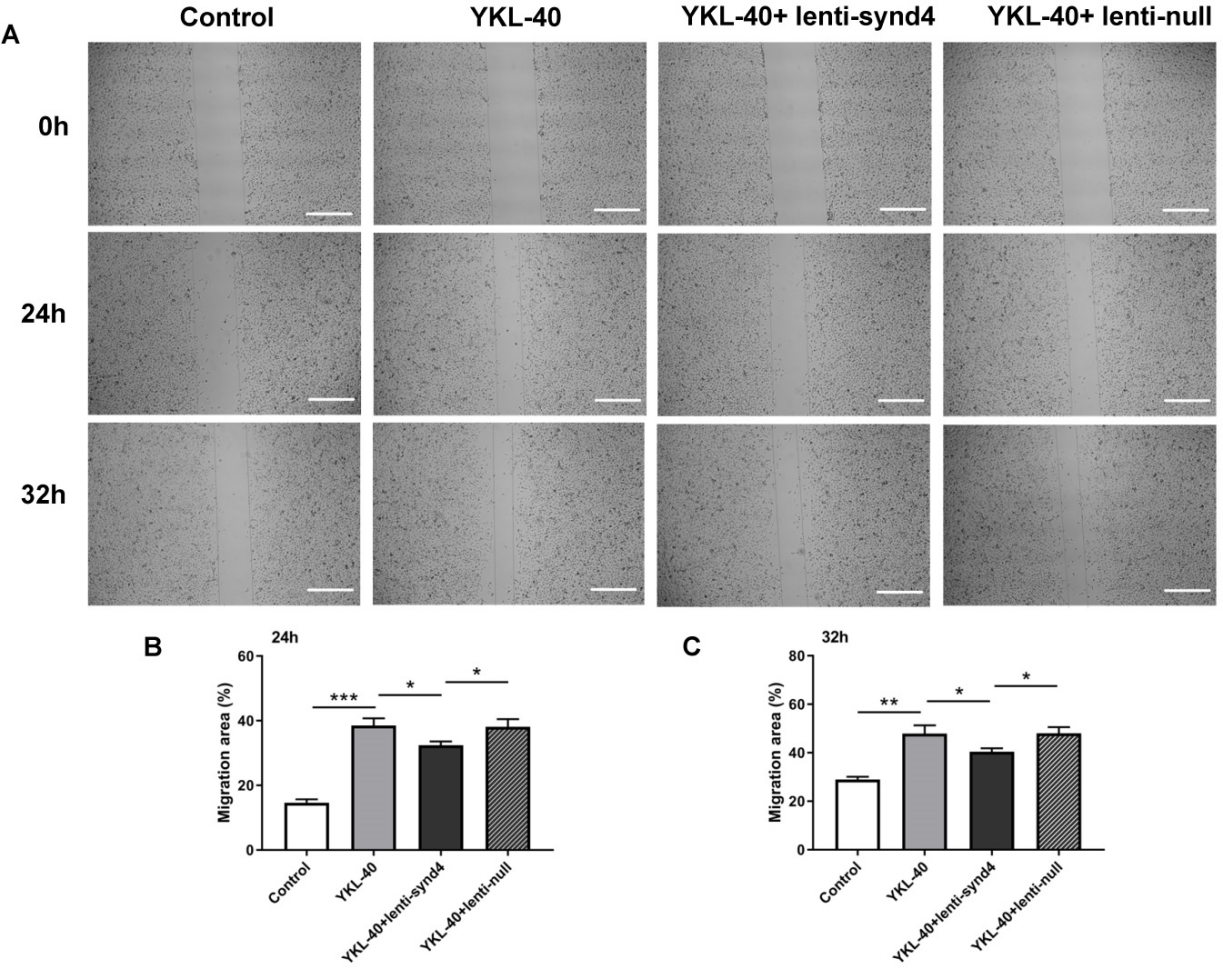
** The effect of lenti-synd4 shRNAs on the migration of HUVECs treated with YKL-40 at 24 hours and 32 hours.** The representative photomicrographs of wound healing assay for control, YKL-40 group, YKL-40+lenti-synd4 shRNAs group and YKL-40+ lenti-null group (**A**). Photos were taken at 24 hours and 32 hours. The relative migration rate was calculated according to the ratio of (initial wound area-terminal wound area)/initial wound area (**B-C**). Data were presented as mean ± S.D. (n=3 per group).*** *p*<0.001, ** *p*<0.01 and *p<0.05 (the scale bar=200um, 40×magnification).

Wound healing assays for evaluating migration of HUVECs were displayed in Figure 3A-C. We found that YKL-40 at concentration of 100 ng/ml remarkably prompted the migration of HUVECs compared with control at 24 hours (p<0.001), and the difference between 100 ng/ml YKL-40 group and control were moderately decreased at 32 hours (p<0.01). Meanwhile, lenti-synd4 shRNAs group significantly decreased the migration rate of HUVECs stimulated with 100 ng/ml YKL-40 after 24 hours and 32 hours (both p<0.05).

### The effect of lenti-synd4 shRNAs on the tube formation of HUVECs treated with YKL-40

**Figure 4:**
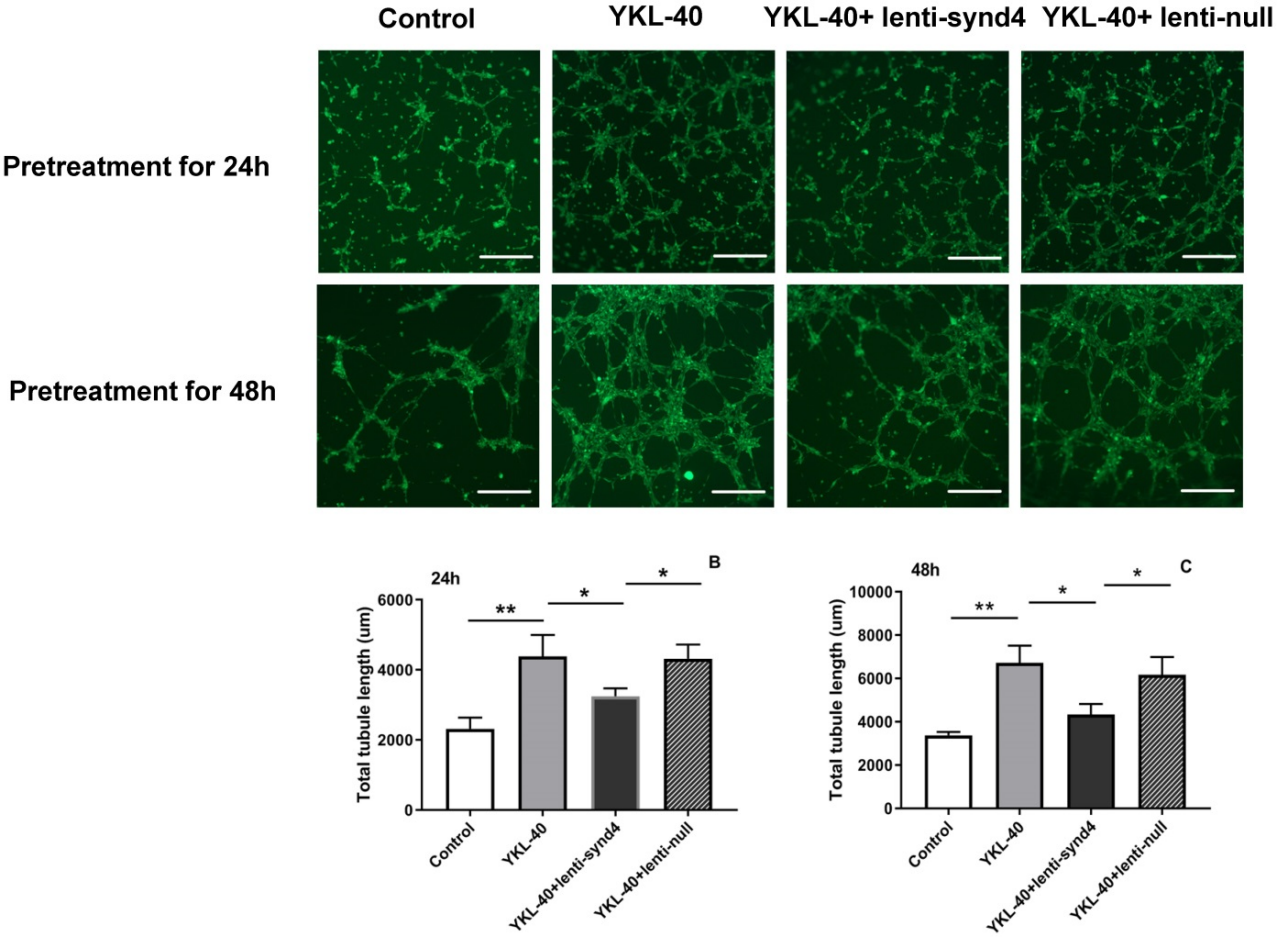
** The effect of lenti-synd4 shRNAs on the tube formation of HUVECs pretreated with YKL-40 for 24 hours and 48 hours.** Representative microscopic images of the tube formation in different groups at different time (**A**). The comparison of angiogenic ability among four groups was calculated according to total tubule length after HUVECs were pretreated with corresponding medium for 24 hours and 48 hours, respectively, (**B-C**). Results were expressed as the mean ± S.D. (n=3 per group). **p<0.01 and *p<0.05 (the scale bar=200um, 40×magnification).

YKL-40 was used to verify its effect on the tube formation in HUVECs. As shown in Figure 4A-C, compared with the control, the total tubule length of HUVECs was remarkably increased after HUVECs were cultured with 100 ng/mL YKL-40 for 24 hours and 48 hours (both p<0.01). We also found that HUVECs transfected with lenti-synd4 shRNAs decreased total tubule length mediated by YKL-40 at 24 hours and at 48 h (both p<0.05). In addition, we further evaluated the differences about the number of nodes, tubules and junctions in respective groups. As a result, the number of nodes (536.7±58.7 vs 157.0±16.7, p<0.001), the number of tubules (175.3±7.0 vs 81.3±9.7, p<0.001) and the number of junctions (176.7±15.6 vs 56.3±11.7, p<0.001) in YKL-40 group was more increased than those in control at 24 hours; and the number of nodes (398.7±25.9 vs 536.7±58.7, p=0.02), tubules (126.3±6.7 vs 175.3±7.0, p=0.001) and junctions (117.7±4.2 vs 176.7±15.6, p=0.003) in group of YKL-40+ lenti-synd4 shRNAs was significantly decreased compared with YKL-40 group. With the extension of culture time, lenti-synd4 shRNAs also remarkably decreased the number of nodes (919.7±36.9 vs 1647.3±83.7, p<0.001), the number of tubules (165.7±14.6 vs 242.7±30.9, p=0.018) and the number of junctions (272.0±21.2 vs 536.3±38.5, p<0.001) in HUVECs pretreated with YKL-40 for 48 hours. The above results showed the effects of YKL-40 on angiogenesis of HUVECs were partly inhibited by downregulating syndecan-4.

### Lenti-synd4 shRNAs inhibited PKCα and ERK1/2 signal pathways in HUVECs cultured with YKL-40

Activation of PKCα and ERK1/2 is regarded as two main signaling pathways for migration and proliferation of endothelial cells. Therefore, we investigated the effects of YKL-40 on the expression of PKCα and ratio of p-ERK1/2 to ERK1/2. The Western blotting analysis showed that after being rectified by control, the levels of PKCα (2.05±0.09 vs 1.52±0.02, p<0.01) and relative value of p-ERK1/2 to ERK1/2 (2.60±0.33 vs 1.97±0.12, p<0.05) between YKL-40 group and YKL-40+lenti-synd4 shRNAs group were significantly different at 24 hours (Figure 4A, C & D). As expected, the protein levels of PKCα (3.22±0.29 vs 2.07±0.23, p<0.01) and p-ERK1/2 to ERK1/2 (2.56±0.19 vs 1.90±0.06, p<0.01) at 48 hours were also higher in YKL-40 group than those in YKL-40 combined with knockdown of syndecan-4, which were shown in Figure 4B, 4E & F.

## Discussion

Recently, YKL-40 also named chitinase-3-like-1 was considered as a multifunctional cytokine which is known to influence inflammation and proliferation in the atherosclerotic lesions 1,17. In clinical investigations, Several studies demonstrated that elevated serum YKL-40 levels were independently associated with the presence and extent of coronary artery disease and even higher YKL-40 levels were observed in patients with acute myocardial infarction 5,6. Moreover, increased concentration of serum YKL-40 was closely related to all-cause as well as cardiovascular mortality in an elderly population 7. Our previous study found that elevated serum YKL-40 was associated with atherosclerotic lesion progression according to angiographic analysis 18. The above studies showed that YKL-40 was possibly associated with unstable plaques. However, the concrete mechanism of YKL-40 on atherosclerosis was still unclear. In our present study, we demonstrated that YKL-40 promoted migration and tube formation of HUVECs, and the role of YKL-40 in angiogenesis of HUVECs was partly mediated by syndecan-4.

Increasing evidence suggests that angiogenesis in the atherosclerotic lesions is an important contributor to unstable plaques 3,19. The neovessels in atherosclerotic plaques are often immature and fragile due to incompleteness of basement membrane, permitting inflammatory cells to infiltrate into the plaques; On the other hand, inflammatory state is a strong inducer for inflammatory cells, such as monocytes/macrophages, releasing pro-angiogenic cytokines which facilitate the initiation and development of angiogenesis 20,21. It was reported that YKL-40 modulated the morphology of vascular endothelial cells by promoting the formation of branching tubules, which showed that YKL-40 have a role in promoting angiogenesis 22. Recently, it has been extensively demonstrated that YKL-40 accelerated tumor angiogenesis and YKL-40 was a potential modulator of inflammatory tumor microenvironment 23,24. The effects of YKL-40 on angiogenesis involved several signaling pathways in various studies, such as focal adhesion kinase (FAK)/protein kinase B (AKT) 25,26. Another study indicated that signaling pathway of mitogen-activated protein kinase (MAPK) also played a critical role in angiogenesis stimulated by YKL-40, and syndecan-1 may be as one of the membrane receptors for YKL-40 13.

Syndecans are a small family of four transmembrane proteoglycans, and they have a similar molecular construction including an N-terminal ectodomain, single transmembrane locus and C-terminal cytoplasmic domain 27. The importance of syndecans is highlighted by an ability to interact with a series of ligands, including extracellular matrix glycoproteins, growth factors, morphogens, and cytokines which are important regulators of regeneration 10. Syndecan-4 is one of the members of syndecans, which are endowed with multiple role including migration, proliferation and homeostasis 28. Unlike other syndecans, syndecan-4 has a widespread tissue distribution. It is demonstrated that syndecan-4 is abundantly expressed in endothelial cells 14,16, and it also plays an important role in the development of angiogenesis 29. Delivery exogenous syndecan-4 could improve angiogenic therapy for ischemia in diabetes; on the contrary, decreasing the expression of syndecan-4 on the cell surface remarkably inhibited the adhesion and migration of endothelial progenitor cells 30,31. In the present study, we found that YKL-40 prompted the migration and tube formation of HUVECs, and downregulation of syndecan-4 inhibited angiogenic potential of HUVECs stimulated by YKL-40. Hence, our study showed that syndecan-4 was possibly a mediator for YKL-40-induced angiogenesis.

Protein kinase C (PKC) and extracellular regulated protein kinases 1/2 (ERK1/2) are main signal pathways involved in the regulation of proliferation, differentiation, cell migration, adhesion and apoptosis 32-34. It has been shown that the syndecan-4 KKXXXKK motif of V region combined with phosphotidylinositol (4,5)-bisphosphate promotes the stabilization of syndecan-4 dimers 35,36. Moreover, this motif is an important bonding location for protein kinase C-alpha (PKCα). Syndecan-4 interacts with PKCα and this interaction regulates localization of PKCα into the cytoskeleton, resulting in a sustained PKC activity 37. As one of mitogen-activated protein kinase (MAPK) family members, the activation of ERK1/2 has also been reported in the angiogenesis induced by syndecan-4 38. In the current study, we likewise demonstrated that YKL-40 promoted the angiogenesis associated with the activation of PKCα and ERK1/2 signal pathways, and inhibiting the gene production of syndecan-4 in HUVECs with lenti-synd4 shRNAs significantly down-regulated the expression of PKCα and ratio of p-ERK1/2 to ERK.

## Conclusion

Our study indicated that the effects of YKL-40 on migration and tube formation of HUVECs were partly regulated by syndecan-4 through PKCα and ERK1/2 signal pathways. It is also clear that YKL-40 is a potential cytokine in promoting angiogenesis and is regarded as a prognostic factor for cardiovascular diseases in previous studies. However, we could not make a definite conclusion that YKL-40 was directly associated with vulnerable atherosclerotic plaque formation and plaque rupture according to current study. In order to verify the hypothesis of YKL-40 promoting unstable atherosclerotic plaque, constructing suitable animal experiments to clarify this mechanism is indispensable in the following studies.

## Figures and Tables

**Figure 5:**
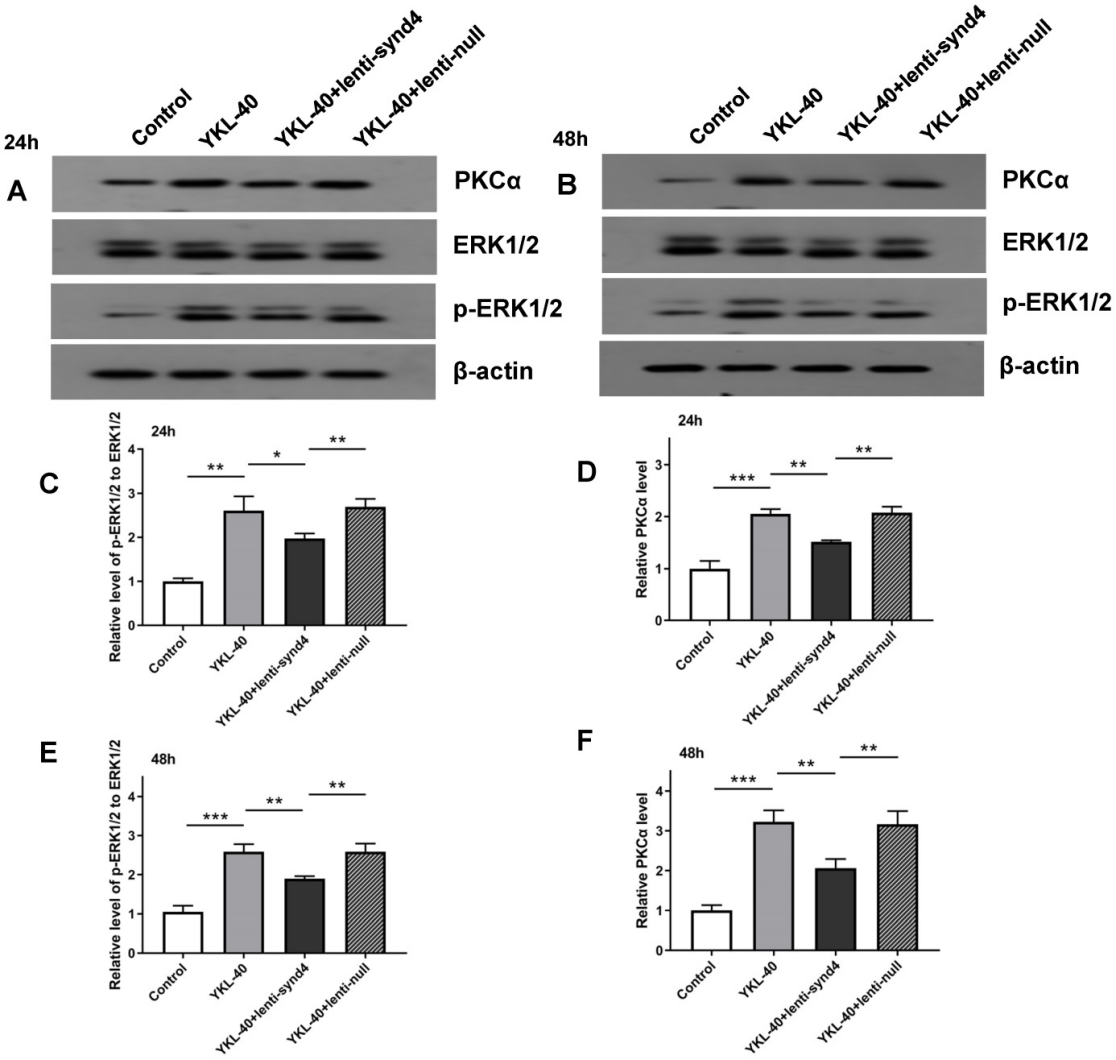
** The protein expression levels of PKCα and p-ERK1/2 to ERK1/2 in HUVECs cultured with YKL-40 were inhibited by lenti-synd4 shRNAs.** The protein expression of PKCα and p-ERK1/2 to ERK1/2 in different groups were determined by western blotting analysis at 24 hours (**A, C-D**) and 48 hours (**B, E-F**). Results were expressed as the mean ± S.D. (n=3 per group). *** *p*<0.001, ** *p*<0.01 and *p<0.05.

**Table 1:** The primer sequences for syndecans and GAPDH

Name	Primer	Sequence
Syndecan-1	Forward	5'-AAGATATCACCTTGTCACAGCA-3'
	Reverse	5'-GTTCTGGAGACGTGGGAATAG-3'
Syndecan-2	Forward	5'-GCTGATGAGGATGTAGAGAGTC-3'
	Reverse	5'-GTATATTCAGCGTCGTGGTTTC-3'
Syndecan-3	Forward	5'-ACCCCAACTCCAGAGACCTT-3'
	Reverse	5'-CCCACAGCTACCACCTCATT-3'
Syndecan-4	Forward	5'-CTTGGTGCCTCTAGATAACCAT-3'
	Reverse	5'-GACACATCCTCACTCTCTTCAA-3'
GAPDH	Forward	5'-AACGTGTCAGTGGTGGACCTG-3'
	Reverse	5'-AGTGGGTGTCGCTGTTGAAGT-3'

Abbreviation: GAPDH: glyceraldehyde-3-phosphate dehydrogenase.
